# Induction of detrusor underactivity by extensive vascular endothelial damages of iliac arteries in a rat model and its pathophysiology in the genetic levels

**DOI:** 10.1038/s41598-019-52811-4

**Published:** 2019-11-08

**Authors:** Myong Kim, Hwan Yeul Yu, Hyein Ju, Jung Hyun Shin, Aram Kim, Jaehoon Lee, Chae-Min Ryu, HongDuck Yun, Seungun Lee, Jisun Lim, Jinbeom Heo, Dong-Myung Shin, Myung-Soo Choo

**Affiliations:** 1 Department of Urology, Asan Medical Center, University of Ulsan College of Medicine, Seoul, 05505 Republic of Korea; 2 Department of Biomedical Sciences, Asan Medical Center, University of Ulsan College of Medicine, Seoul, 05505 Republic of Korea; 3 Department of Physiology, Asan Medical Center, University of Ulsan College of Medicine, Seoul, 05505 Republic of Korea; 4 Department of Urology, Konkuk University Hospital, Konkuk University School of Medicine, Seoul, 05030 Republic of Korea

**Keywords:** Bladder disease, Bladder

## Abstract

We tried to establish a reliable detrusor underactivity (DUA) rat model and to investigate pathophysiology of chronic bladder ischemia (CBI) on voiding behavior and bladder function. Adult male rats were divided into five groups. The arterial injury (AI) groups (AI-10, AI-20, AI-30) underwent vascular endothelial damage (VED) of bilateral iliac arteries (with 10, 20, and 30 bilateral repetitions of injury, respectively) and received a 1.25% cholesterol diet. The sham group underwent sham operation and received the same diet. Controls received a regular diet. After 8 weeks, all rats underwent unanesthetized cystometrogram. Bladder tissues were processed for organ bath investigation, immunohistochemistry staining, and genome-wide gene expression analysis. Awake cystometry analysis showed that frequency of voiding contractions and micturition pressure were lower in the AI-30 group than in sham group (p < 0.01). Contractile responses to various stimuli were lower in AI-20 and AI-30 groups (both p < 0.001). In the AI-20 and AI-30 groups, atherosclerotic occlusion in the iliac arteries, tissue inflammation, fibrosis, denervation, and apoptosis of bladder muscle were prominent compared to the sham. Mechanistically, the expression of purinergic receptor P2X-1 was reduced in the AI-30 group, and the genome-wide gene expression analysis revealed that genes related to IL-17 and HIF-1 signaling pathways including INF-γ receptor-1 and C-X-C motif chemokine ligand-2 were upregulated in the CBI-induced DUA rat model. A rat model of progressive VED successfully induced DUA. Abnormal tissue inflammation, fibrosis, denervation, and bladder muscle tissue apoptosis may be involved in CBI-induced DUA pathophysiology.

## Introduction

Detrusor underactivity (DUA) is defined as reduced detrusor contraction strength and/or duration, resulting in prolonged bladder emptying and/or failure to achieve complete bladder emptying within a normal time span^1^. DUA is a frustrating diagnosis for clinicians as well as patients since pharmacological treatment has suboptimal efficacy^2,3^. In patients refractory to oral pharmacotherapy, alternative treatment option is lifelong clean intermittent catheterization (CIC) or indwelling urethral catheter with the attendant risk of urethral injury, recurrent urinary tract infection, occasional bladder perforation, and possible deteriorating renal function^4,5^. In addition, the socioeconomic and psychological burdens of lifelong CIC must be considered^6,7^. Annually, neurogenic bladder patients have on average 16 office and 0.5 emergency room visits in the United States^7^.

The prevalence of urodynamically confirmed DUA in elderly patients with lower urinary tract symptoms (LUTS) is approximately 28.0% (40.2% in males and 13.3% in females), and the prevalence of DUA increases with age^8^. Vascular endothelial damage (VED) also occurs during the human aging process and is an independent risk factor for atherosclerosis and hypertension^9^. Pelvic arterial insufficiency, a common clinical problem in elderly population, may lead to impaired lower urinary tract perfusion and has an important role in voiding dysfunction, such as DUA or detrusor overactivity (DO).

Animal models reproducing DUA are scarce. Studies in a rabbit model of chronic bladder ischemia (CBI) suggested that moderate ischemia causes DO^10^, while severe ischemia causes bladder DUA^11^. Similar findings were also observed in a rat model^12^. Nomiya *et al*. reported that progressive VED may lead to bladder DUA in rats^12^. However, previous CBI-induced DUA rat models have some limitations. First, the pattern of the voiding function induced in the CBI rat model was so varied as to be unpredictable. The previous CBI rat model induced DUA^12,13^, but it also induced overactive bladder in the same study settings^14^. Second, previous studies have utilized artificial agents such as a nitric oxide synthase inhibitor (L-NAME) in the rat model to enhance the vascular intimal changes^14^. Lastly, the pathophysiology of CBI-induced DUA was not fully elucidated in previous studies^12,13^.

Considering the above, we attempted to establish a more reliable DUA rat model. We hypothesized that sufficient physical damage for VED, without any enhancers, is sufficient to induce DUA. We investigated the effects of the severity of CBI on voiding behavior and bladder function in rat models. Moreover, genome-wide gene expression profiling was carried out to identify molecular pathways strongly associated with the pathogenesis of CBI-induced DUA.

## Results

### Cystometrogram analysis

**Figure 1:**
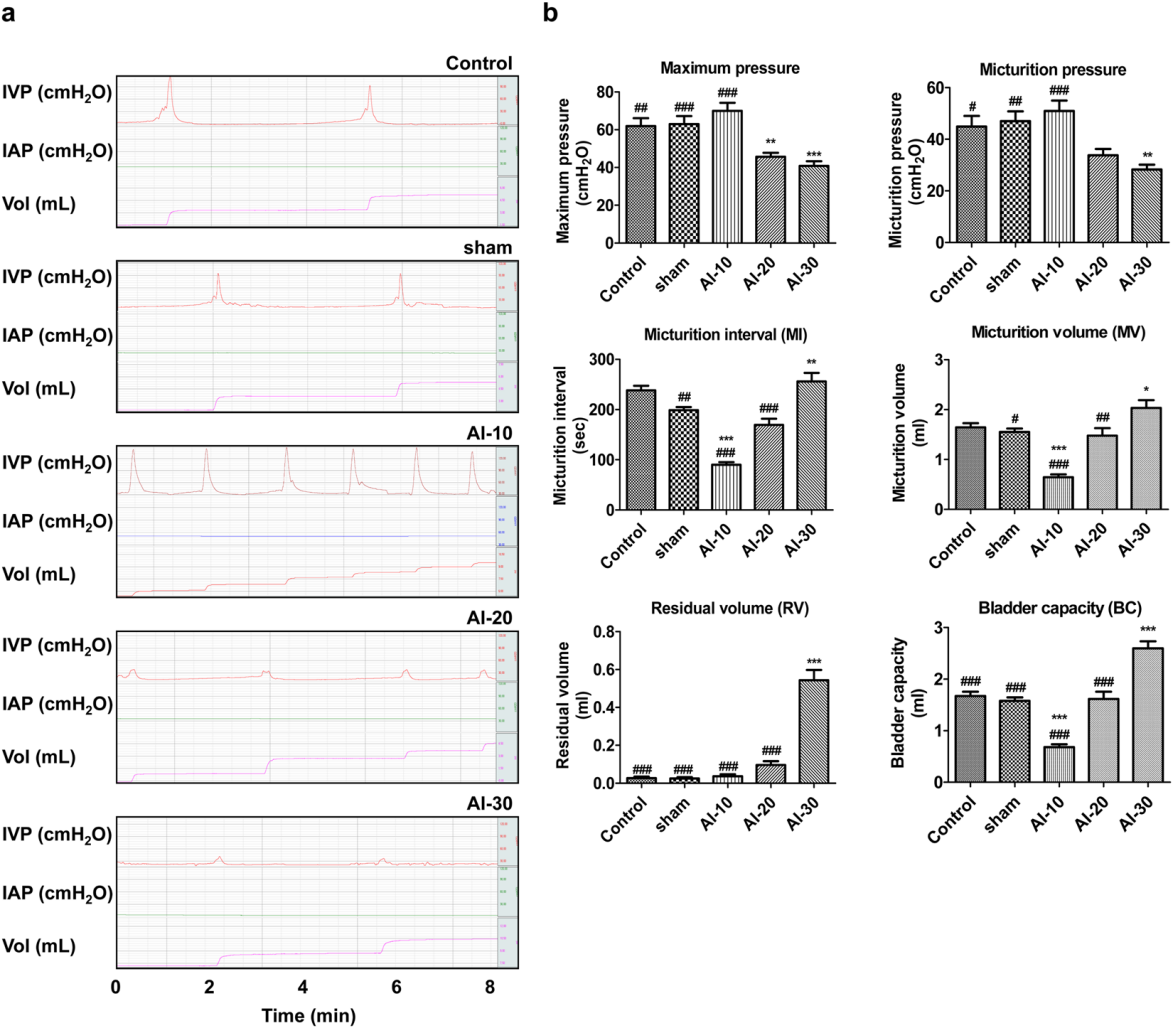
Effect of severity of chronic bladder ischemia on bladder voiding function (**a**,**b**) Representative awake cystometry results; and (**b**) quantitative bladder voiding data of animals in the indicated groups at 8 weeks after iliac artery injury. Control n = 15; sham n = 17; AI-10 n = 15; AI-20 n = 18; AI-30 n = 16. All data are presented as mean ± SEM, *p < 0.05, **p < 0.01, ***p < 0.001 compared to the sham group; #p < 0.05, ##p < 0.01, ###p < 0.001 compared to the AI-30 group. Source data with exact p-values and number of replicates can be found in the Supplementary Dataset.

To examine whether the severity of VED of bilateral iliac arteries could affect the bladder voiding functions, 10, 20, and 30 repetitions of arterial injury (AI), followed by a 1.25% high-cholesterol diet for 8 weeks were applied to the rat model (Supplementary Figure 1). We performed awake filling and voiding cystometrogram studies, which allow long-term evaluation of bladder voiding function in free-moving animals^15–17^. The changes in the dynamics of voiding function assessed by the awake cystometrogram were dramatically different according to the degree of vascular injury (Fig. 1). In the AI-10 group, voiding contractions were more frequently observed (Fig. 1a) and the micturition interval (MI) was significantly shorter than that of sham group (p < 0.001; Fig. 1b). However, maximum pressure and micturition pressure were not significantly reduced compared to the sham group. The micturition volume (MV) of the AI-10 group was significantly smaller than that of the sham group (p < 0.001), whereas the residual volume (RV) of the AI-10 group was not significantly increased (Fig. 1b).

Conversely, in the AI-20 and AI-30 groups, in maximum pressure and micturition pressure were also reduced compared to the sham group. In particular, in the AI-30 group, voiding contractions were less frequent (Fig. 1a) and the MI of the AI-30 groups was significantly longer than that of the sham group (p < 0.01; Fig. 1b). In addition, both the maximum and micturition pressures were significantly reduced (p < 0.001 and p < 0.01), and the RV and bladder capacity (BC) were significantly increased compared to the sham group (p < 0.001; Fig. 1b). Collectively, these results indicated that the degree of VED altered bladder functions ranging from DO to DUA and also suggested that progressive VED tended to induce DUA rather than DO.

### Organ bath study

**Figure 2:**
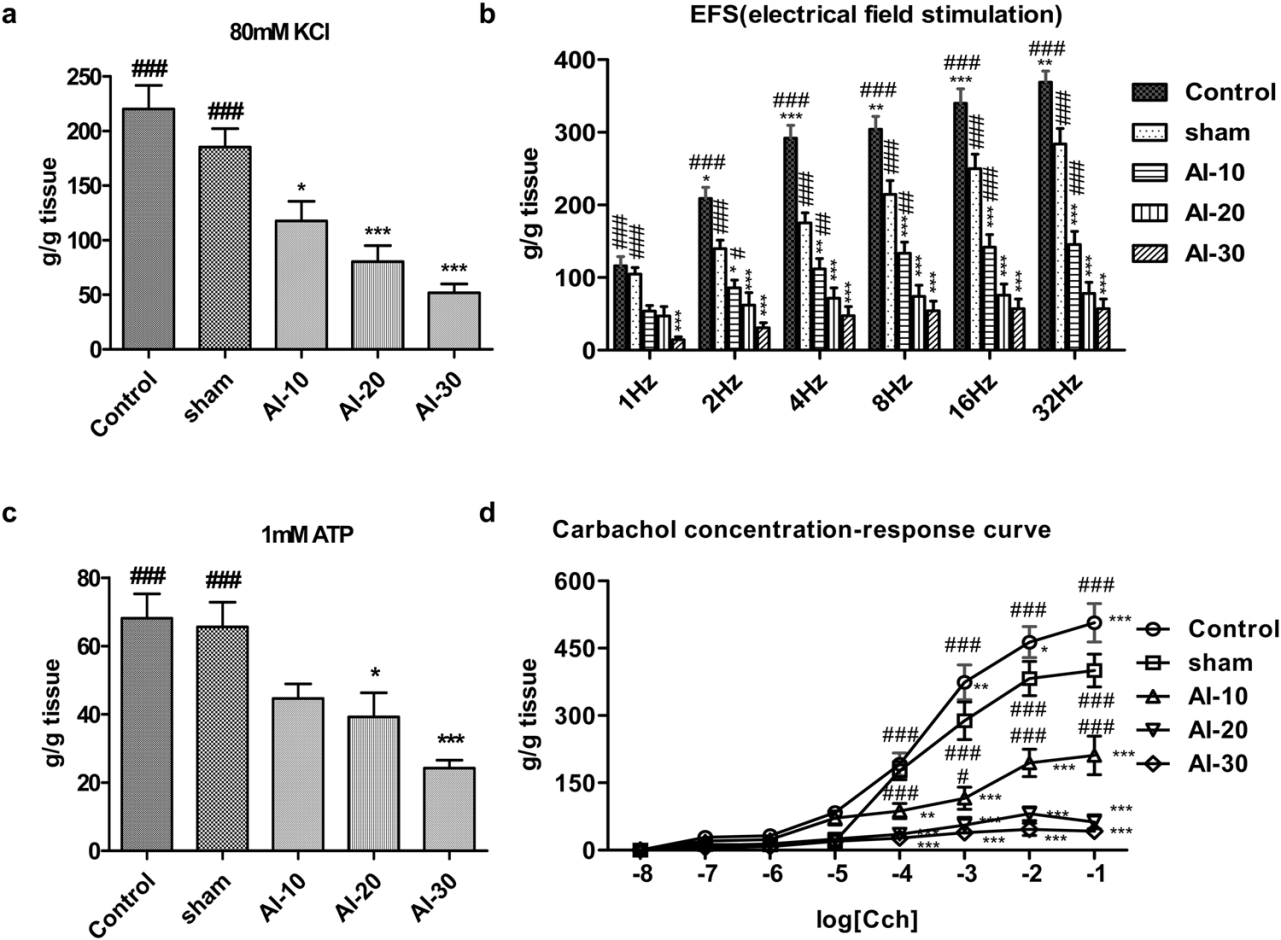
Functional loss in contraction of bladder smooth muscle by the chronic bladder ischemia (**a,d**) Organ bath analysis for contractile response of bladder muscle in the indicated groups; (**a**) Contractile response to 80 mM KCl; (**b**) frequency–response to EFS; (**c**) contractile response to 1 mM ATP; and (**d**) concentration–response curve to carbachol. Control n = 7 (14 bladder strips); sham n = 7 (14 bladder strips); AI-10 n = 6 (12 bladder strips); AI-20 n = 7 (14 bladder strips); AI-30 n = 7 (14 bladder strips). All data are presented as mean ± SEM, *p < 0.05, **p < 0.01, ***p < 0.001 compared to the sham group; #p < 0.05, ##p < 0.01, ###p < 0.001 compared to the AI-30 group. Source data with exact p-values and number of replicates can be found in the Supplementary Dataset.

Thereafter, to assess changes in the overall contractile response, bladder strips of all groups were initially exposed to KCl (Fig. 2a). The mean contractile responses to KCl in the AI-10, AI-20, and AI-30 groups decreased according to the degree of vascular injury and were significantly lower than in the sham group (p < 0.05 in the AI-10 group and p < 0.001 in the AI-20 and AI-30 groups; Fig. 2a). Contractile responses induced by electrical field stimulation (EFS) were significantly reduced according to the degree of vascular injury at all frequencies (1 Hz to 32 Hz) (p < 0.001; Fig. 2b). The contractile responses to 1 mM ATP in the AI-20, and AI-30 groups were significantly lower than in the sham group (p < 0.05 and p < 0.001), and were lower according to the degree of vascular injury (Fig. 2c). The response to carbachol in the AI-10, AI-20, and AI-30 groups at the concentrations of 1 nM to 1 mM were significantly lower than in the sham group (p < 0.001) and the reductions in response to carbachol were dependent on the degree of vascular injury (Fig. 2d).

### Histologic examination

#### Common iliac arterial wall thickness

**Figure 3:**
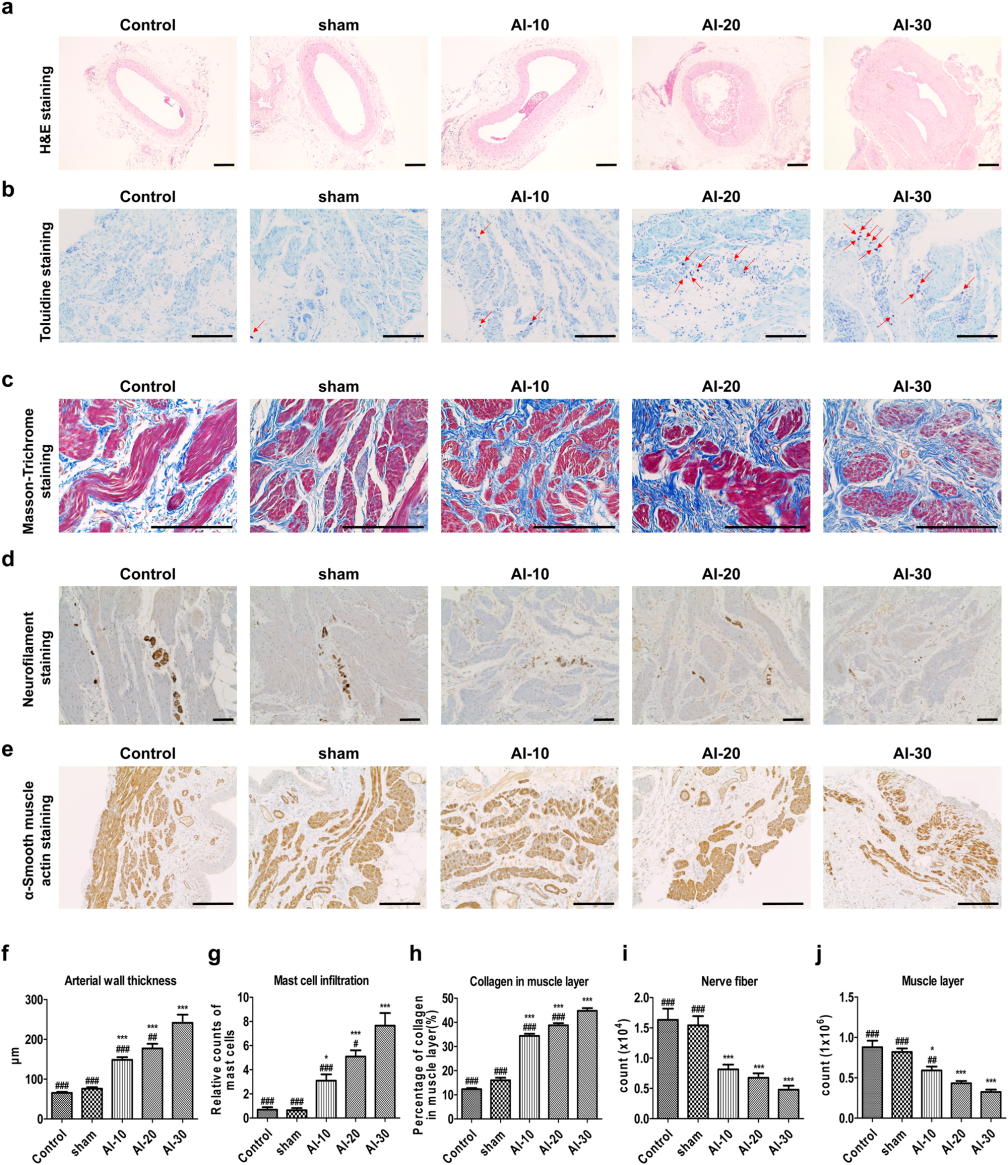
Histologic features of bladder tissue damaged by chronic bladder ischemia. (**a**) Hematoxylin and eosin (H&E) staining in the iliac artery tissues (magnification ×100, scale bar = 200 μm) of animals in the indicated groups after 8 weeks with iliac artery injury; (**b**) Toluidine blue staining (magnification ×200, scale bar = 200 μm); and (**c**) Masson’s trichrome staining (magnification ×400, scale bar = 200 μm) in the bladder tissues of the indicated groups; (**d**,**e**) Immuno-histochemical staining of neurofilament 200 (**d**, magnification ×100, scale bar = 200 μm) and α-smooth muscle actin (**e**, magnification × 400, scale bar = 200 μm) in the bladder tissues; (**f,j**) Quantification data of the aforementioned histological results. Data are presented as mean ± SEM (n = 20 except n = 14 in (**f**). *p < 0.05, **p < 0.01, ***p < 0.001 compared to the sham group; ^#^p < 0.05, ^##^p < 0.01, ^###^p < 0.001 compared to the AI-30 group according to a one-way ANOVA with the Bonferroni post-hoc test. Source data with exact p-values and number of replicates can be found in the Supplementary Dataset.

Hematoxylin and eosin staining of iliac artery cross-sections from the AI groups demonstrated obvious arterial wall thickening (Fig. 3a,f). In particular, the AI-30 group showed prominent neointimal formation compared to the sham and other AI groups (Fig. 3a). The average iliac artery wall thicknesses in the AI-10, AI-20, and AI-30 groups were significantly greater than in the sham group (all p < 0.001) and showed a tendency for greater thickness with higher degree of vascular injury (Fig. 3f).

#### Histo-pathophysiological analysis

Compared to the sham group, the bladder tissues in the AI-10, AI-20, and AI-30 groups demonstrated significant increase in the infiltration of toluidine blue-stained mast cells (Fig. 3b,g). Masson’s trichrome staining of bladder tissue revealed an increased percent of collagen in the muscle layer in the AI-10, AI-20, and AI-30 groups compared to the sham group (Fig. 3c). The AI-10, AI-20, and AI-30 groups differed significantly from the sham-operated animals, respectively (all p < 0.001; Fig. 3h). Moreover, immunohistochemical (IHC) analysis indicated that significant denervation in the bladder muscle layer of AI-10, AI-20, and AI-30 groups (all p < 0.001) was observed on NE-100 staining (Fig. 3d,i). In addition, immunostaining with anti-alpha smooth muscle actin (α-SMA) antibody revealed that the bladder muscle layer in the AI-10 (p < 0.05), AI-20 (p < 0.001), and the AI-30 (p < 0.001) groups was significantly more atrophic than in the sham group (Fig. 3e) and the severity of this effect was dependent on the degree of vascular injury (Fig. 3j).

**Figure 4:**
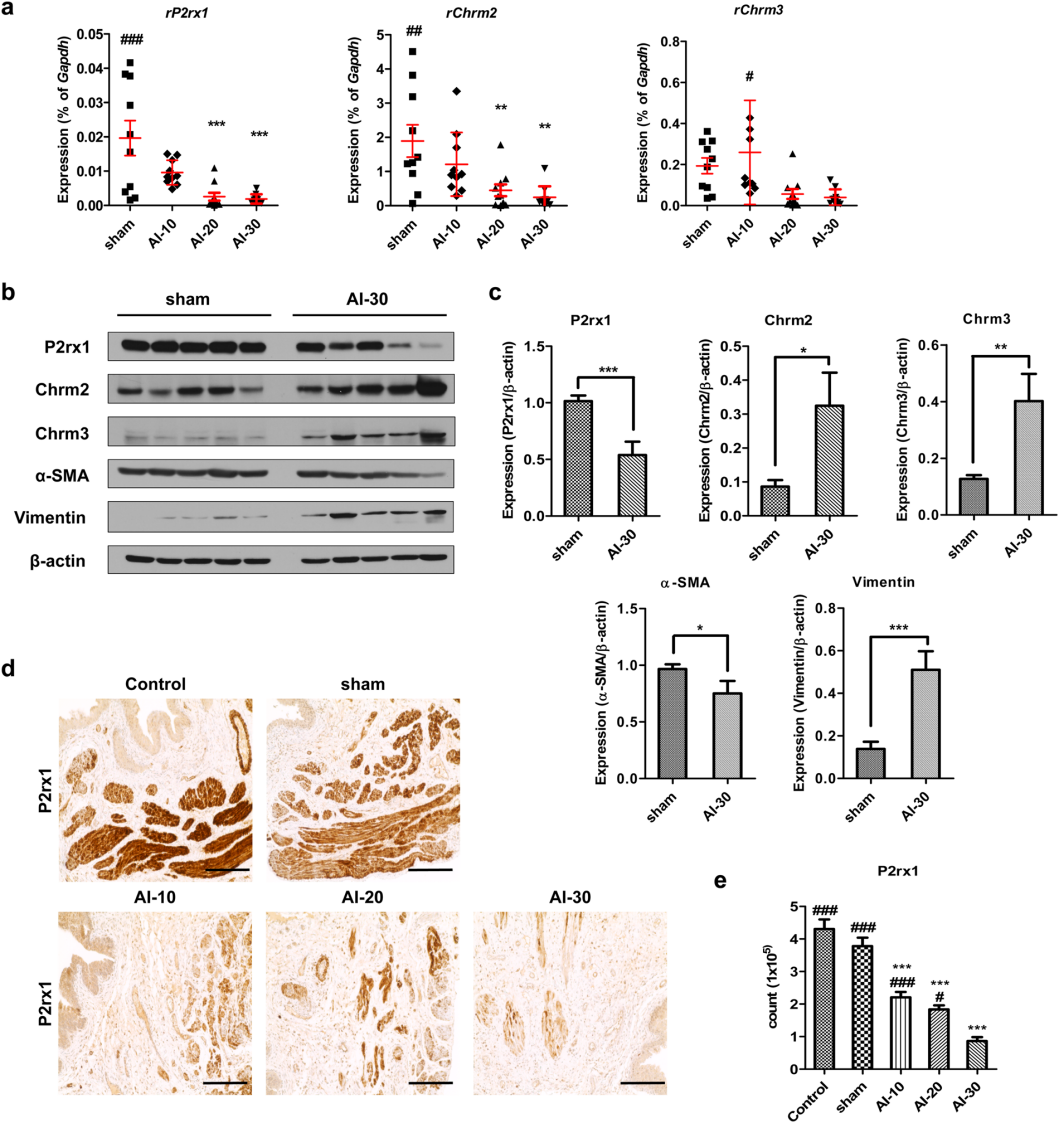
Repression of P2rx1 by progressive vascular endothelial damage. (**a**–**c**) RQ-PCR (**a**) and western blot (**b**,**c**) analysis of the expressions of the cholinergic receptor muscarinic 2 (*Chrm2*), cholinergic receptor muscarinic 3 (*Chrm3*), and purinergic receptor P2X 1 (*P2rx1*) genes in the bladders of rats in the indicated groups. Expression levels of the indicated transcripts are presented as % *Gapdh*. For western blot analysis, β-actin was used as a loading control, and the expression levels of the indicated proteins were normalized to the β-actin value. (**d**,**e**) Representative images (**d**) and quantification analysis (**e**) of the immunohistochemical staining for the P2rx1 protein (original magnification ×200, scale bar = 200 μm). All quantification results are presented as mean ± SEM. *p < 0.05, **p < 0.01, and ***p < 0.001, compared to the sham group; ^###^p < 0.05, ^###^p < 0.01, ^###^p < 0.001, compared with the AI-30 group according to the unpaired *t*-test (**c**) or one-way analysis of variance with the Bonferroni post hoc test. (**a**–**e**) Source data with exact p-values and number of replicates can be found in the Supplementary Dataset.

In the organ bath study, we observed reduced contractility in bladder tissues of the AI-10, AI-20, and AI-30 groups (Fig. 2). When we examined the expression levels of genes associated with mediating contractile stimuli of the detrusor muscle, cholinergic receptor muscarinic-2 (*Chrm2*), cholinergic receptor muscarinic-3 (*Chrm3*), and purinergic receptor P2X-1 (*P2rx1*) transcript levels were significantly reduced as degree of vascular injury increased (Fig. 4a). The western blot analysis revealed that among the receptors, only the P2rx1 protein was significantly downregulated in the bladder tissues of animals in the AI-30 group (Fig. 4b,c). The IHC analysis of the bladder tissues further validated the decrease in the P2rx1 protein expression level by progressive VED (Fig. 4d,e). The bladder tissues of the animals in the AI-30 group were characterized by decreased α-SMA protein expression level, which correlated with the organ bath and IHC results (Fig. 4b,c). However, the level of vimentin, a marker protein for stromal cells, was increased in the AI-30 group.

#### Apoptosis analysis

**Figure 5:**
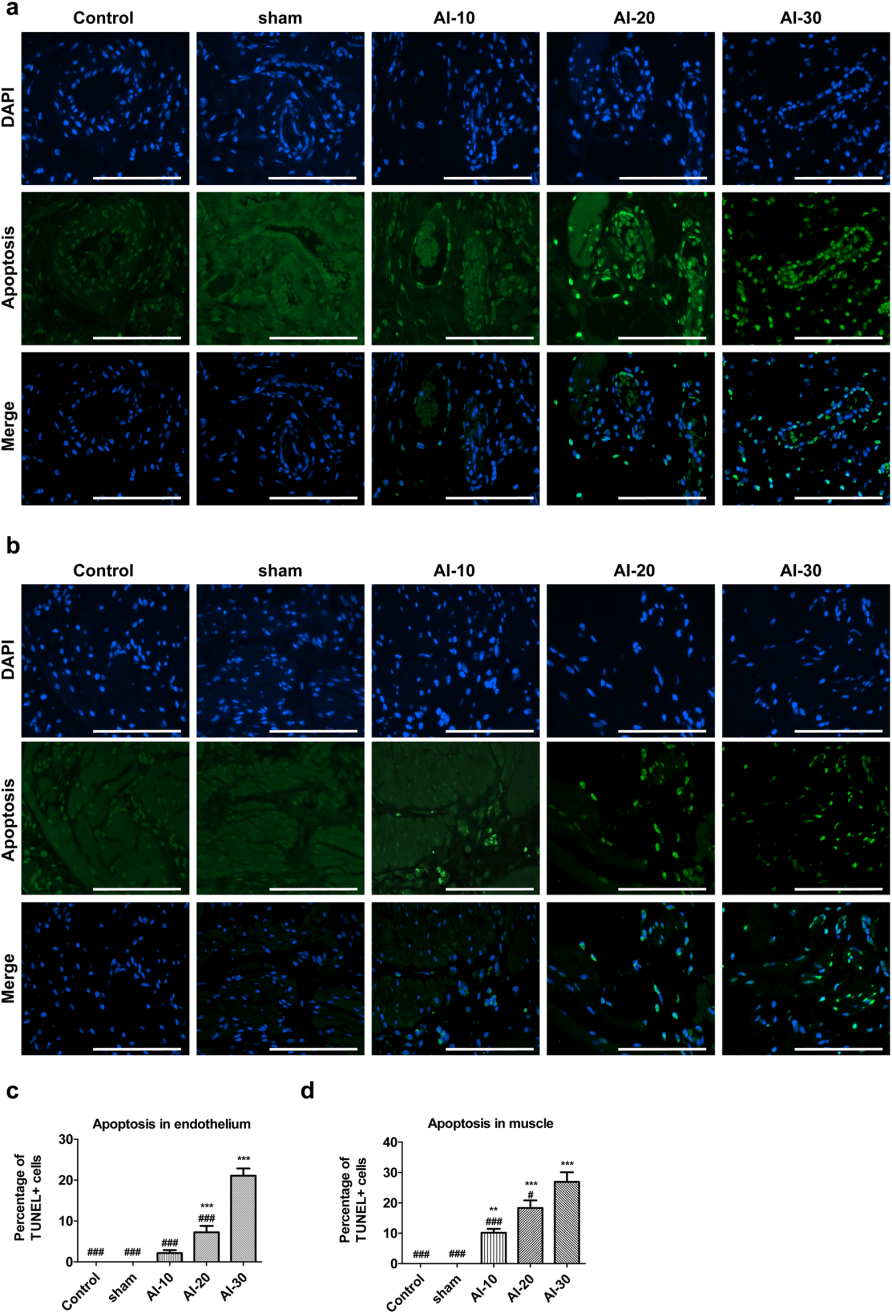
Increased apoptosis in the bladder tissues by the chronic bladder ischemia. Representative images of staining to detect apoptotic cells (green) in the bladder endothelium. (**a**) and muscle layer (**b**) of animals in the rat of indicated groups (magnification ×400, scale bar = 20 μm); (**c**,**d**) Quantification of the staining results. Percentage of apoptotic cells was quantified by calculating the ratio of apoptotic cells (TUNEL-positive) to total cells (DAPI-stained nuclei). All data are presented as mean ± SEM (n = 20). *p < 0.05, **p < 0.01, ***p < 0.001 compared to the sham group; ^#^p < 0.05, ^##^p < 0.01, ^###^p < 0.001 compared to the AI-30 group according to a one-way ANOVA with the Bonferroni post-hoc test. Source data with exact p-values and number of replicates can be found in the Supplementary Dataset.

TUNEL staining of bladder tissues revealed a significantly higher percentage of apoptosis in the endothelium (Fig. 5a,c) and muscle layer (Fig. 5b,d) in the AI-20 and AI-30 groups compared with the sham group. The AI-10 group tended to have more frequent apoptosis in the endothelial and in muscle layer (Fig. 5a,b). The difference in apoptosis between the AI-10 and sham groups failed to reach statistical significance in the endothelial layer (Fig. 5c), however the frequency of TUNEL stained apoptotic cells was significantly increased in the muscle layer of the AI-10 group (Fig. 5d).

Taken together, these histological examinations suggested that a high degree of atherosclerotic occlusion by progressive VED (AI-20 and AI-30 groups) induced histological features frequently observed in the bladder of patients with DUA including tissue inflammation, fibrosis, denervation, muscular degeneration, and increased apoptosis of the bladder muscle tissue.

#### Gene expression study

**Figure 6:**
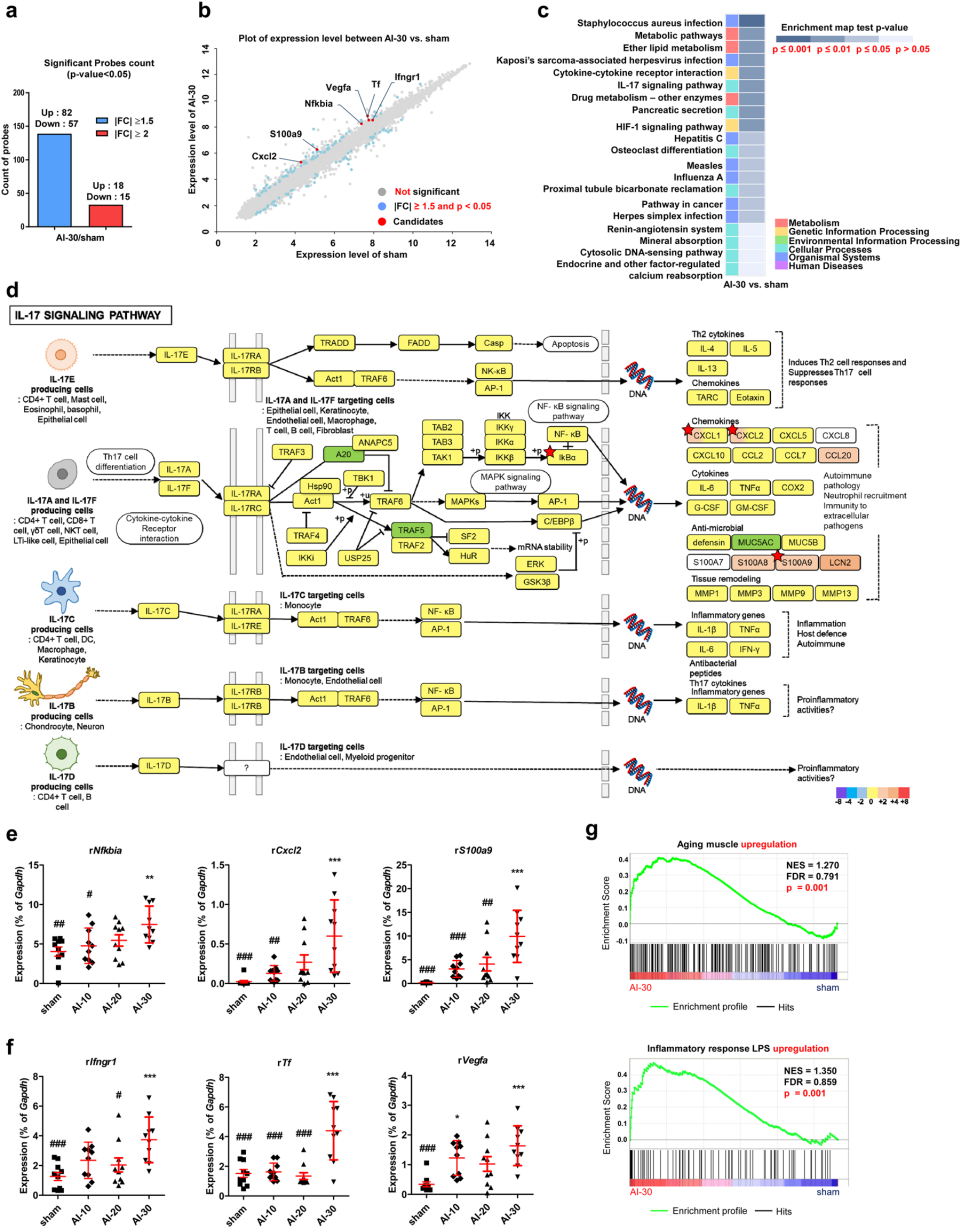
Genome-wide gene expression profiles of bladder tissues with AI-30 injury. **(a**) Count of significant up- and down-regulation probes count in transcriptome analysis of bladder tissues in comparison between AI-30 and sham groups; (**b**) Scatter plot analysis of the transcriptomes. Significant genes based on cut-off values with |FC| ≥1.5 and p-value < 0.05 were marked as blue dots and the putative candidates for biomarkers were marked as red dots; (**c**) KEGG pathway analysis using significant genes based on cut-off values with |FC| ≥1.5 and p-value ≤0.05 in comparison between AI-30 and sham groups. The enrichment map test p-value and gene ontology of each pathway were shown in upper and low right corners, respectively; (**d**) KEGG pathway mapping of the IL-17 signaling pathway. Annotated genes in transcriptome analysis were mapped against KEGG pathway maps (www.kegg.jp/kegg/kegg1.html) using a KEGG mapper tool (http://www.kegg.jp/kegg/tool/map_pathway2.html)^39–41^. Candidate genes with significance was marked with red asterisks; (**e**,**f**) RQ-PCR analysis of genes which were significantly represented in the IL-17 signaling pathway (**e**) and HIF-1 signaling pathway (**f**) on KEGG pathway analysis. Expression is presented as % *Gapdh* and shown as dot plot of mean ± SEM (n = 10; *p < 0.05, **p < 0.01, and ***p < 0.001, compared with the sham group; ^###^p < 0.05, ^###^p < 0.01, and ^###^p < 0.001, compared with the AI-30 group); (**f**) GSEA enrichment plots of gene sets representing aging muscle upregulation gene set (top panel)^18^ and genes involved in inflammatory response LPS upregulation gene set (bottom panel)^19^ in a comparison of AI-30 versus sham transcriptomes; GSEA, gene set enrichment analysis; NES, normalized enrichment score; FDR, false discovery rate. Source data with exact p-values and number of replicates can be found in the Supplementary Dataset.

To obtain molecular insight into DUA induced by ischemic vascular injury, we compared the genome-wide gene expression profiles of bladder tissues of sham and AI-30-treated groups. Based on cut-off values with a fold change of more than 1.5 and p-value < 0.05, approximately 139 genes were differentially expressed genes (DEGs) and the majority (82 genes) were up-regulated in the AI-30 group (Fig. 6a,b). KEGG pathway analysis indicated that DEGs between AI-30 and the sham groups were significantly associated with interleukin-17 (IL-17) and hypoxia inducible factor-1 (HIF-1) pathways (Fig. 6c and Supplementary Figure 2). In particular, the up-regulation of NFKB Inhibitor-alpha (*Nfkbia*), C-X-C motif chemokine ligand-2 (*Cxcl2*), and S100 calcium binding protein-A9 (*S100a9*) was significant in the IL-17 pathway (Fig. 6d) and interferon gamma receptor-1 (*Ifngr1*), transferrin (*Tf*), and vascular endothelial growth factor-A (*Vegfa*) were considered key contributing genes for the significant differences observed in HIF-1 pathways following AI-30 injury (Supplementary Figure 2). Immunofluorescent staining revealed the increase of Hif-1α proteins in the bladder tissues of AI-30 group (Supplementary Figure 3), further indicating the significance of HIF-1 pathway.

Real-time quantitative PCR (RQ-PCR) analysis validated that AI-30 injury specifically increased the transcription of the aforementioned genes associated with the IL-17 (Fig. 6e) and HIF-1 (Fig. 6f) pathways. In addition, we identified an additional 25 genes belonging to distinct biological pathways altered in the response to AI-30 injury (Table 1). These include phospholipase A2 group IIA (*Pla2g2a*) and colony stimulating factor-3 receptor (*Csf3r*), involved in ether lipid metabolism or cytokine-cytokine receptor interaction pathways, respectively (Supplementary Figure 4). In addition, progressive VED injury significantly increased the transcription of complement factor-H (*Cfh*), C2 complement (*C2*), Fc fragment of immunoglobulin G receptor-IIb (*Fcgr2b*), and interferon-induced protein with tetratricopeptide repeats 1B-like (*Ifit1bl*), genes related to pathogen infection processes (Supplementary Figure 4).

**Table 1:** List of genes significantly changed by progressive vascular endothelial damage (AI-30 group).

Gene Symbol	mRNA Accession	AI-30/sham fold change	AI-30/sham p-value	Map Name
*S100a9*	NM_053587	2.27	0.04	IL-17 signaling pathway
*Tf*	NM_001013110	2.24	0.04	HIF-1 signaling pathway
*Csf3r*	NM_001106685	2.19	0.01	Cytokine-cytokine receptor interaction
				Pathways in cancer
*Hmgcs2*	NM_173094	2.14	0.03	Metabolic pathways
*Pla2g2a*	NM_031598	2.09	0.00	Ether lipid metabolism
				Metabolic pathways
				Pancreatic secretion
*Cxcl2*	NM_053647	2.02	0.05	Cytokine-cytokine receptor interaction
				IL-17 signaling pathway
				Kaposi’s sarcoma-associated herpesvirus infection
*Ifit1bl*	XM_001079971	1.98	0.04	Hepatitis C
				Herpes simplex infection
*Cfh*	NM_130409	1.98	0.05	Staphylococcus aureus infection
*Car2*	NM_019291	1.95	0.04	Proximal tubule bicarbonate reclamation
				Pancreatic secretion
*Slc4a4*	NM_053424	1.86	0.01	Proximal tubule bicarbonate reclamation
				Pancreatic secretion
*Nfkbia*	NM_001105720	1.83	0.04	Osteoclast differentiation
				IL-17 signaling pathway
				Hepatitis C
				Measles
				Influenza A
				Kaposi’s sarcoma-associated herpesvirus infection
				Herpes simplex infection
				Pathways in cancer
*C2*	NM_172222	1.80	0.01	Staphylococcus aureus infection
*Ces2g*	NM_001106175	1.79	0.04	Drug metabolism - other enzymes
*Fcgr2b*	NM_175756	1.78	0.01	Osteoclast differentiation
				Staphylococcus aureus infection
				Measles
*Vegfa*	NM_001110333	1.71	0.04	Cytokine-cytokine receptor interaction
				HIF-1 signaling pathway
				Kaposi’s sarcoma-associated herpesvirus infection
				Pathways in cancer
*Ptafr*	NM_053321	1.68	0.04	Staphylococcus aureus infection
*Il33*	NM_001014166	1.59	0.04	Influenza A
*B3gnt5*	NM_053932	1.59	0.01	Metabolic pathways
*Ifngr1*	NM_053783	1.51	0.01	Cytokine-cytokine receptor interaction
				HIF-1 signaling pathway
				Osteoclast differentiation
				Measles
				Influenza A
				Kaposi’s sarcoma-associated herpesvirus infection
				Herpes simplex infection
				Pathways in cancer
*Pla2g7*	NM_001009353	1.51	0.03	Ether lipid metabolism
				Metabolic pathways
				Pancreatic secretion
*Agps*	ENSRNOT00000002111	−1.50	0.00	Ether lipid metabolism
*Cldn23*	NM_001033062	−1.65	0.01	Hepatitis C
*Sc5d*	NM_053642	−1.82	0.05	Metabolic pathways
*Cmpk1*	NM_001025655	−2.19	0.05	Drug metabolism - other enzymes
				Metabolic pathways
*Cda*	NM_001108688	−2.91	0.02	Drug metabolism - other enzymes
				Metabolic pathways

We further analyzed the biological pathways enriched in the transcriptome of bladder tissues of the AI-30 group using Gene Set Enrichment Analysis (GSEA). The gene sets associated with aging muscle^18^ and the LPS-mediated inflammatory response^19^ were characteristically enriched in the expression profiles of the AI-30 group compared to the sham-operated group (Fig. 6g). On examining ischemic VED injury, GSEA analysis indicated that gene sets involved in reactive oxygen species^20^ and oxidative phosphorylation (contributed by KEGG pathway) were positively represented in the transcriptome of the AI-30 group in comparison to the sham group (Supplementary Figure 5).

## Discussion

In the present study, we identified that progressive VED of bilateral iliac arteries, without the contribution of any of enhancers, successfully induced changes in bladder function similar to DUA in a rat model. These findings suggest that CBI may be responsible for a major pathogenetic mechanism observed in DUA. The understanding of the pathogenesis of DUA remains uncertain. However, it is likely to be multifactorial^21^. It is recognized that detrusor contractility diminishes with aging although not all individuals develop clinically relevant DUA^8^. It is presumed that multifactorial conditions may cause DUA. In some patients without any pathological causes, an age-related decrease in detrusor contractility might be primarily responsible for “idiopathic” DUA and this might explain why the gene set related to aging muscle was characteristically enriched by progressive VED (Fig. 6g). In other patients, relevant conditions such as diabetes mellitus, neural injury, or bladder outlet obstruction may lead to “secondary” DUA^21^.

Using previously-established rat models, it has been suggested that CBI induced by VED combined with a high-cholesterol diet can lead to functional and structural changes in the bladder^12–14^. However, as mentioned above, changes observed in voiding patterns induced in previous CBI rat models have also been varied^13,14,22^. Nomiya *et al*. first reported that balloon endothelial injury of the iliac arteries (performed 10 times on each side) with 8 weeks of a high-cholesterol diet induced DO manifested as an increase in voiding frequency^14^, and proposed that oxidative stress and inflammation may be key factors in the development of DO^22^. Conversely, the same study group also reported that the CBI rat model produced fibrosis and a reduction in the number of nerves innervating the bladder, which led to decreased bladder contractility in the same setting (10 times of both iliac arterial injury combined with a high-cholesterol diet)^13^.

The results of our study also demonstrated a wide spectrum of changes in the bladder function according to the degree of VED. In the AI-10 group, voiding contractions were more frequently observed (Fig. 1a) and the MI was significantly shorter than that of the sham group (Fig. 1b). These results were similar to those of the former study by Nomiya *et al*.^14^, which reproduced the DO.

Conversely, more progressive vascular damage resulted in different results. In the AI-20 and AI-30 groups, voiding contractions tended to be less frequent (Fig. 1a), and the MI of AI-30 group were significantly longer than that of the sham group (Fig. 1b). In particular, in the AI-30 group, micturition pressure was significantly reduced and RV was significantly increased compared to the sham group (Fig. 1b). Moreover, our results from organ bath studies confirmed that contractile responses to various stimulations decreased proportionately with the degree of vascular injury (Fig. 2). These results implied that some degree of bladder ischemia induced by VED could result in the development of DO manifested as an increase in voiding frequency, however, more progressive VED and ischemia may reduce the bladder contractility and therefore result in DUA manifested as an emptying failure.

These findings are in agreement with a more recent study by Nomiya *et al*.^12^, which also reported that progressive VED induced DUA in a rat model. In that study, rats were exposed to L-NAME (3 mg/mL dissolved in drinking water) to enhance the morphological vascular intimal changes, in addition to the VED (10 times to both iliac arteries) and a high-cholesterol diet. The iliac arteries of the injury/L-NAME groups showed more prominent neointimal formation and luminal occlusion. Furthermore, the RV in the injury/L-NAME group tended to be increased, while bladder contractility was significantly decreased compared to those in controls^12^. Although, the negative effects of L-NAME on blood flow via the vascular intimal change have been demonstrated in previous rat model studies^12,23,24^, L-NAME, may also exaggerate the effects of hypoxic damage via generation of nitrogen species^25^. Therefore, chronic exposure to L-NAME may have a direct effect on bladder function in rat models^26^.

For these reasons, we simply hypothesized that sufficient physical damage for VED itself is enough to induce DUA. Our results clearly showed that sufficient VED (AI-30) successfully caused prominent arterial wall thickening and neointimal formation compared with the sham-operated and other AI groups (Fig. 3a). Moreover, our AI-30 group without any exposure to the artificial enhancer drug, reproduced similar changes in bladder functions as DUA, as observed in the injury/L-NAME groups of a previous study^12^. In particular, in the previous model by Nomiya *et al*., the injury/L-NAME group did not demonstrate any significant decrease in micturition pressure on cystometry assessment^12^, our result in the AI-30 group showed that maximum and micturition pressure were significantly reduced compared to the sham group (Fig. 1b). Therefore, we suggest that adequate VED alone is sufficient and more efficient for inducing the DUA in the rat model.

The pathophysiology of CBI-induced DUA has not been fully elucidated by previous studies^12,13^. These studies have suggested that collagen deposition in the muscle layer^12,13^ and significantly fewer nerves in the bladder wall^13^ caused by oxidative stress from chronic bladder ischemia may be the possible causes for decreased contractile function based on histological evaluations. However, these previous studies did not consistently report the results of histological changes of the CBI-induced DUA rat model^12,13^. Our results clearly showed that progressive VED induced mast cell infiltration (Fig. 3b,g) and collagen deposition in the muscle layer (Fig. 3c,h). Moreover, significantly fewer nerves in the bladder wall of AI-10, AI-20, and AI-30 groups were also observed (Fig. 3d,i). These results are in agreement with previous studies^12,13^. In addition, we also observed a significant atrophy of the bladder detrusor muscle in the AI groups (Fig. 3e,j). Our results based on immunofluorescence staining suggest that this significant muscle atrophy is directly caused by the apoptosis of bladder muscle cells (Fig. 5). These results imply that oxidative stress from progressive chronic bladder ischemia causes the inflammation of bladder tissue, and therefore it induces fibrosis and denervation of the bladder muscle layer, moreover it also induces apoptosis of bladder muscle tissues. In this regard, the transcriptome of bladder tissue of the AI-30 group was characteristically enriched by gene sets associated with oxidative stress and inflammatory responses (Supplementary Figure 5). Thus, further study is required to identify a driver gene(s) responsible for these histological changes and to advance our understanding of the pathogenesis of impaired detrusor contractility induced by the progressive VED.

Regarding the possible pathophysiology of DUA induced by progressive VED at the genetic level little is known. Some researchers have proposed the transforming growth factor-β1 (TGF-β1) pathway as a possible pathophysiological mechanism for DUA^13^. This suggestion was supported by the results from another researcher group, which demonstrated that the expression of TGF-β1 RNA and protein levels were increased in bladder tissue and that collagen fibers in the bladder muscle layer were increased in the rabbit CBI model^27^. Our genome-wide gene expression analysis showed that genes (e.g. *Nfkbia, Cxcl2*, and *S100a9*) related to the IL-17 and HIF-1 pathways (Fig. 6e,f) were particularly upregulated by progressive VED. In several chronic tissue injuries models including bleomycin-treated mice, the production of T cell IL-17 is a key mediator of tissue inflammation and subsequent fibrosis through the activation of TGF-β signaling^28^. Furthermore, the abnormal expression of *Nfkbia, Cxcl2*, and *S100a9* has been associated with the pathogenesis of a wide range of chronic inflammation and age-related tissue degeneration disorders^29–31^. Therefore, as a future study, we will attempt not only to identify the crucial gene(s) involved in DUA pathogenesis, but also to develop an associated novel therapeutic strategy for treating these intractable bladder voiding dysfunction disorders.

In particular, CXCL2, also known as macrophage inflammatory protein-2 was associated with a number of hypoxic tissue injuries including mouse hindlimb post-ischemia model^32^, post-ischemic myocardium^33^, hypoxia-induced liver injury^34^, and hypoxia treatment in diabetic rats^35^, suggesting the possible cross-talk between the IL-17 and HIF-1 pathways, characteristic to ischemic VED in this study. In the response to these tissue injuries, CXCL2 is produced by a variety of cell types including as macrophages, monocytes, epithelial cells, and endothelial cells^36^. Therefore, further investigation of the regulatory mechanisms and cellular source of CXCL2 induction may be helpful not only to advance our understanding of the pathogenesis of DUA, but also to develop CXCL2-targeted therapeutic strategies for this intractable disorder.

In the present study, a rat model of progressive VED without any the use of artificial enhancers successfully induced DUA. Our data suggest that oxidative stress from progressive VED followed by tissue inflammation, fibrosis, denervation, and apoptosis of the bladder muscle tissue may represent underlying mechanisms for CBI-induced DUA. At the genetic level, IL-17 and HIF-1 signaling pathways including INF-γ receptor-1 and *Cxcl2* seem to be the key modes of action, which provoke DUA, and might represent helpful treatment targets for DUA in the future.

## Methods

### Ethics statement and study approval

All animal experiments were approved and performed in accordance with guidelines and regulations of the Institutional Animal Care and Use Committee of the University of Ulsan College of Medicine (IACUC-2018-12-145).

### Study design

Male 16-week old Sprague–Dawley rats were divided into control (n = 15), sham (n = 17), and AI groups treated with 10 (AI-10; n = 15), 20 (AI-20; n = 18), and 30 AI repetitions (AI-30; n = 16). The AI-10, AI-20, and AI-30 rats were anesthetized with intraperitoneal injection of pentobarbital (25 mg/kg), and a 2-French Fogarty arterial embolectomy catheter (E-060-2F, Edwards Lifesciences, Irvine, CA, USA) was passed through the femoral artery into the common iliac artery. The balloon was inflated and subsequently withdrawn from the common iliac artery to the femoral artery (Supplementary Figure 1), a maneuver repeated 10 times (AI-10 group), 20 times (AI-20 group), and 30 times (AI-30 group) on each side. Sham, AI-10, AI-20, and AI-30 groups received a 1.25% cholesterol diet (D12336, Research Diets). After 8 weeks, all rats underwent unanesthetized cystometrogram analysis. Thereafter, bladder tissues and iliac arteries were processed for organ bath investigations, immunohistochemistry staining, and gene expression analysis. For all experimental settings, animals were randomly allocated according to treatment group, order of injury, and order of cystometry. All cystometric, histological, and gene expression assessments were performed by investigators who were blinded to the treatment groups. Any animals that died unexpectedly by bladder insult or catheter implantation were excluded from the analyses.

### Unanesthetized and unrestrained cystometrogram

The evaluation was performed in the awake state with an unrestrained animal model in metabolic cages. Three days prior to the cystometrogram, catheters were placed in the bladder and intraabdominal cavity to record intravesical pressure (IVP) and intraabdominal pressure (IAP), respectively, as described elsewhere^15–17,37,38^. The urethra was approached using a PE-50 catheter (Clay Adams, Parsippany, NJ) connected to a pressure transducer (Research Grade Blood Pressure Transducer, Harvard Apparatus, Holliston, MA, USA) and a microinjection pump (PHD22/2000 pump, Harvard Apparatus). Voided volumes were recorded by means of a fluid collector connected to a force displacement transducer (Research Grade Isometric Transducer, Harvard Apparatus) as normal saline was infused into the bladder at a rate of 0.4 mL/min. The IVP and IAP were recorded continuously and then analyzed using the Acq Knowledge 3.8.1 software and MP150 data acquisition system (Biopac Systems, Goleta, CA, USA) at a sampling rate of 50 Hz. The mean values from three reproducible voiding cycles using individual animals were used for evaluation. Maximum pressure was defined as the maximum IVP during the micturition cycle. The micturition pressure was defined as the maximum IVP subtracted by IAP during the micturition cycle. The MV was the volume of expelled urine, and RV as the urine volume remaining following voiding. RV was measured by aspiration of residual urine using the PE-50 catheter immediately after urination. Bladder capacity (BC) was estimated by the summation of MV and RV. Cases with accidental errors of the pressure or volume sensors were excluded from the cystometric analysis.

### Organ bath study

Longitudinal strips of the posterior wall of the bladder dome were mounted in organ baths (5 mL) containing Krebs solution and bubbled with 5% CO2 and 95% O2 (37 °C). One hook was suspended from a transducer (type 45196 A; San-ei Instruments, Tokyo, Japan), and the lower hook was fixed to a plastic support leg attached to a micrometer (Mitutoyo, Tokyo, Japan). Each strip was equilibrated unstretched for 40 minutes. A load of 2.0 g was applied to each strip by micrometer adjustment and the load was readjusted to this level 30 minutes later. Changes in the tone of the strips were measured isometrically using force transducers and the data were recorded using LabChart v7.3.8 software and a PowerLab/16sp data acquisition system (AD Instruments, Castle Hill, Australia). Contractions by the KCl (P9333; Sigma-Aldrich, St. Louis, MO, USA; 80 mM), ATP (A2383; Sigma-Aldrich; 1 mM), EFS (1, 2, 4, 8, 16, and 32 Hz), and carbachol (PHR1511; Sigma-Aldrich; 1 nM to 1 mM) were recorded. For EFS stimulation, an electrical pulse (1 millisecond pulse width and 80 V in the bath) was delivered using a stimulator (D-7806, Hugo Sachs Elektronik, Germany) for 5 seconds at increasing frequencies (1, 2, 4, 8, 16 and 32 Hz), followed by intervals of stimulation of 5 minutes. All bladder strips were normalized to weight per 1 g.

### Histologic examination

The iliac artery thickness of each group was quantified. Mast cell and collagen infiltration in muscle layer were assessed using toluidine blue staining (toluidine blue-O, Daejung Chemicals & Metals Co., Gueonggi-do, Korea) and Masson’s trichrome staining (Junsei Chemical, Tokyo, Japan), respectively. The presence of peripheral nerves in the bladder muscle layer was assessed by N,N-dipropyl-2-[4-methoxy-3-(2-phenylethoxy)phenyl] ethylamine staining (SML0631; NE-100, Sigma-Aldrich). In addition, anti-α-SMA antibody staining (ab5694; Rabbit, Abcam) was performed to evaluate the bladder muscular atrophy. Apoptosis of the bladder mucosa and muscle layer was assessed by terminal deoxynucleotidyl transferase dUTP nick-end labeling staining (1 684 795; TUNEL, Roche, Mannheim, Germany) and the nuclei were counterstained with 4’,6-diamino-2-phenylindole (D9542; DAPI, Sigma-Aldrich). Antibodies specific to P2rx1 (APR-001; Alomone labs, Israel) and Hif-1α (ab1, H1alpha67; Abcam, Cambridge, MA, USA) was also used. Quantitative digital image analysis was performed from two randomly chosen representative areas selected from each slide using Image Pro 5.0 software (Media-Cybernetics, Rockville, MD, USA).

### Transcriptome microarray analysis

Total RNA was isolated from the bladder tissues from AI-30 and sham groups using the RNeasy Mini Kit (QIAGEN, Valencia, CA, USA), including treatment with DNase I (QIAGEN). One microgram of total RNA was subjected to analysis using the Affymetrix GeneChip Rat Gene 2.0 ST Array (Affymetrix, Santa Clara, CA, USA). Microarray image data were processed on a GeneChip GCS3000 Scanner and Command Console software (Affymetrix). After importing CEL files of six samples (three independent samples from each group), the data were summarized and normalized using the robust multi-average (RMA) method implemented in the Affymetrix Expression Console Software.

Functional analysis of transcriptomes was performed using KEGG (Kyoto Encyclopedia of Genes and Genome)^39–41^ pathway analysis or GSEA microarray software with default settings, as described previously^42,43^. In the KEGG pathway analysis, 1.5-fold up- or down-regulation with p < 0.05 was defined as the cut-off value for significant change. For GSEA analysis, gene sets were obtained from published literature or filtered from a curated functional gene set (C2) database. The transcriptome data discussed in this study have been deposited in the Gene Expression Omnibus of the NCBI and are accessible under GEO Series accession number GSE122060.

### Gene expression analysis

For the RQ-PCR analysis, reverse transcription of the isolated total RNA was performed using TaqMan reverse transcription reagents (Applied Biosystems) and the expression level of the indicated transcripts was quantified by RQ-PCR with the PikoReal RT-PCR System (Thermo Fisher Scientific, Inc., Waltham, MA, USA) with iQ SYBR Green PCR Master Mix (Bio-Rad, Hercules, CA), as described previously^44,45^. Two randomly chosen areas from each slide or duplicated RQ-PCR assays (n = 10) using five independent animals were used to quantify the digital image or gene expression data.

For the western blot analysis, bladder tissue samples were extracted using the RIPA lysis buffer (Sigma-Aldrich). Protein concentrations in the tissue extracts were quantified using the bicinchoninic acid method (Thermo Scientific). Protein levels were assessed from 50 μg of extracts separated using 10% SDS-PAGE gels by probing with antibodies specific to P2rx1 (APR-001, Alomone Labs), Chrm2 (AMR-002, Alomone Labs,), Chrm3 (AMR-006, Alomone Labs, Israel), α-SMA (ab5694, Rabbit, Abcam), vimentin (sc-6260, Santa Cruz Biotechnology Inc., CA), and β-actin (Sigma-Aldrich). Density of the indicated protein bands was quantified using the ImageJ software (National Institute of Mental Health, Bethesda, MD), and their expression levels were calculated by normalization to the β-actin values. The uncropped western blots are presented as Supplementary Figure 6.

### Statistics

Data are reported as the mean ± standard error of the mean (SEM) and were analyzed using GraphPad Prism 7.0 software (GraphPad Software, La Jolla, CA). Differences and significance were verified by one-way or two-way ANOVA followed by Bonferroni post hoc tests. A p-value < 0.05 was considered as statistically significant.

## Supplementary information

supplementary information

Dataset 1
