# Supplementary material for: Induction of detrusor underactivity by extensive vascular endothelial damages of iliac arteries in a rat model and its pathophysiology in the genetic levels
Source: Sci Rep. 2019 Nov 8;9:16328. doi: 10.1038/s41598-019-52811-4 (PMC6841737; doi:10.1038/s41598-019-52811-4)
Supplement: Supplementary file 1 — supplementary information [file 41598_2019_52811_MOESM1_ESM.docx]

Induction of detrusor underactivity by extensive vascular endothelial damages of iliac arteries in a rat model and its pathophysiology in the genetic levels

Myong Kim^1,§^, Hwan Yeul Yu^1,2,§^, Hyein Ju^2,3,§^, Jung Hyun Shin^1^, Aram Kim^4^, Jaehoon Lee^1^, Chae-Min Ryu^1,2^, HongDuck Yun^2,3^, Seungun Lee^2,3^, Jisun Lim^2,3^, Jinbeom Heo^2,3^, Dong-Myung Shin^2,3,*^, Myung-Soo Choo^1,*^

^1^Department of Urology, ^2^Department of Biomedical Sciences, ^3^Department of Physiology, Asan Medical Center, University of Ulsan College of Medicine, Seoul, 05505, Republic of Korea, ^4^Department of Urology, Konkuk University Hospital, Konkuk University School of Medicine, Seoul, 05030, Republic of Korea

**^§^**These authors contributed equally to this work.

*Co-correspondence:

Myung-Soo Choo, M.D., Ph.D.

Department of Urology, Asan Medical Center, 88 Olympic-ro 43-gil, Songpa-gu, Seoul 05505, Republic of Korea.

Phone: +82230103735, Fax : +8224778928, E-mail: mschoo@amc.seoul.kr

Dong-Myung Shin, Ph.D.

Department of Biomedical Sciences, Asan Medical Center, 88 Olympic-ro 43-gil, Songpa-gu, Seoul 05505, Republic of Korea

Phone: +82230102086, Fax : +82230108493, E-mail: d0shin03@amc.seoul.kr

SUPPLEMENTARY FIGURE LEGENDS


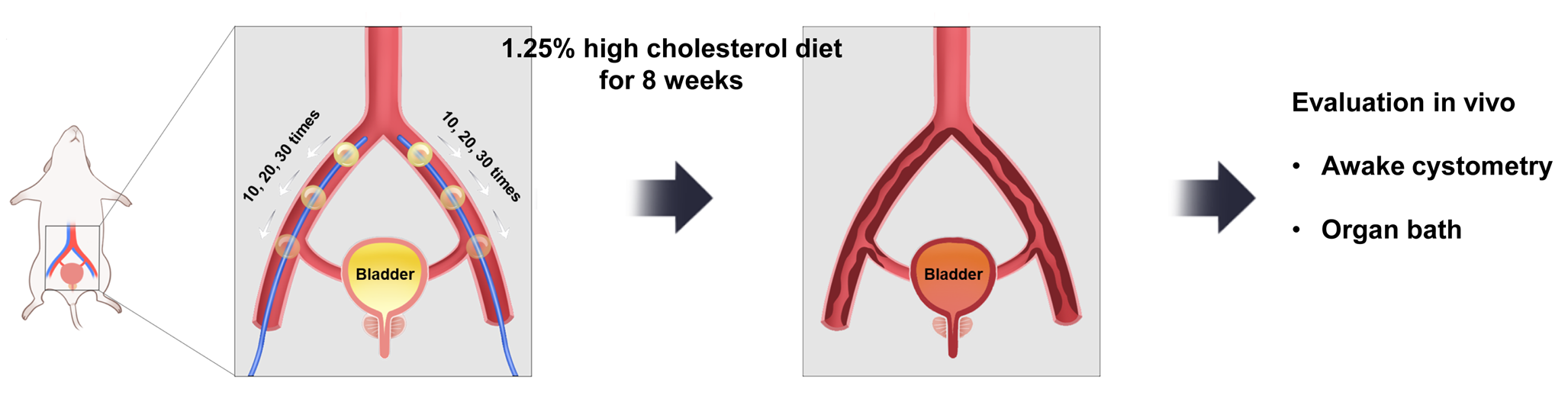


**Supplementary figure 1.** Experiment overview.

In the chronic bladder ischemia (CBI) modeling, vascular endothelial damages (VEDs) of the bilateral iliac arteries of different severities were induced by performing 10, 20, and 30 repetitions of arterial injury (AI), followed by a 1.25% high-cholesterol diet for 8 weeks.

.


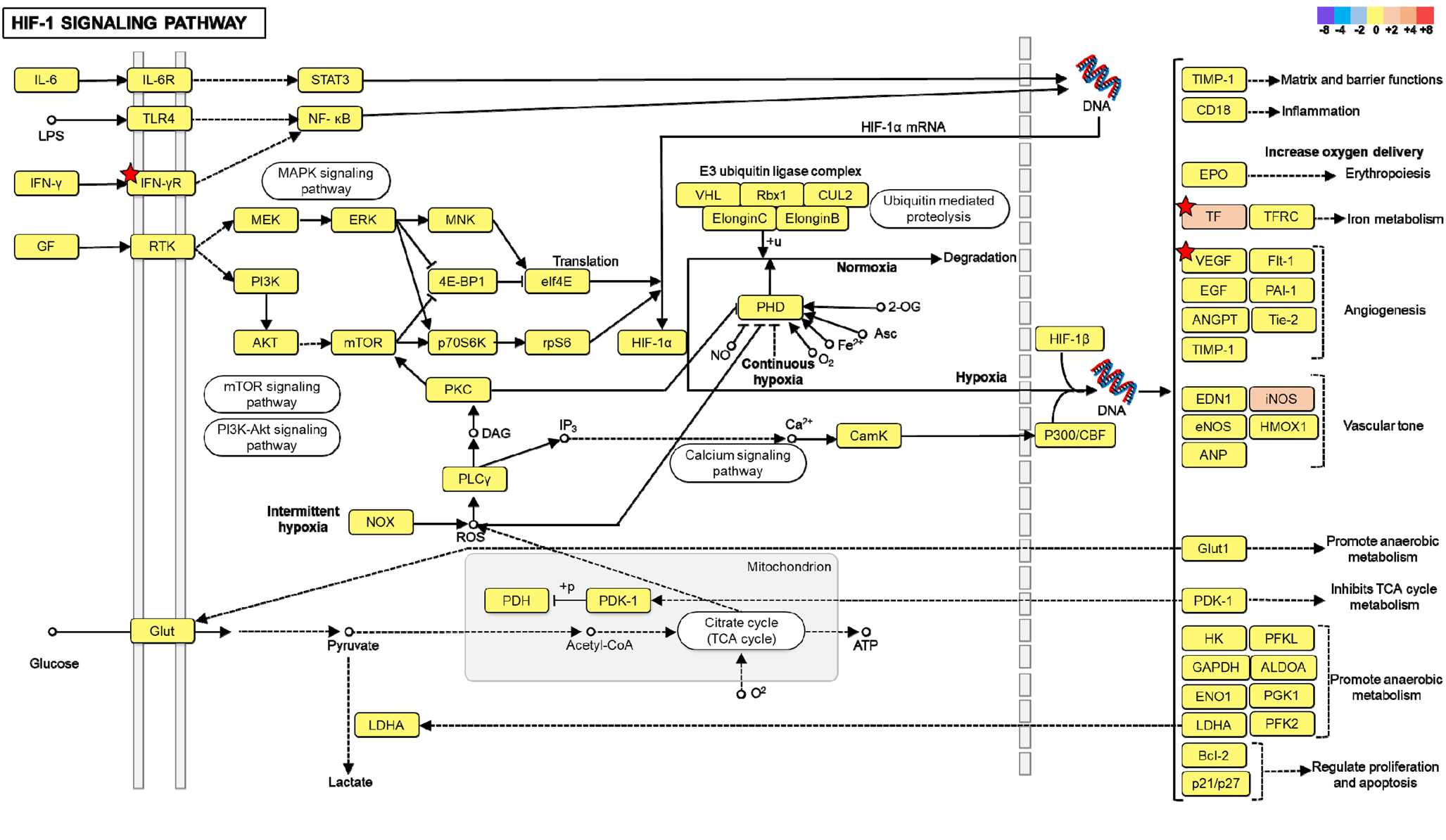


**Supplementary figure 2.** Overview of hypoxia inducible factor-1 (HIF-1) signaling pathway map

KEGG pathway mapping of the HIF-1 signaling pathway in a comparison of AI-30 versus sham transcriptomes. Annotated genes in transcriptome analysis were mapped against KEGG pathway maps ([www.kegg.jp/kegg/kegg1.html](http://www.kegg.jp/kegg/kegg1.html)) using a KEGG mapper tool (<http://www.kegg.jp/kegg/tool/map_pathway2.html>)^39-41^. Candidate genes with significance including TF, VEGF, and IFN-γR were marked with red asterisks.


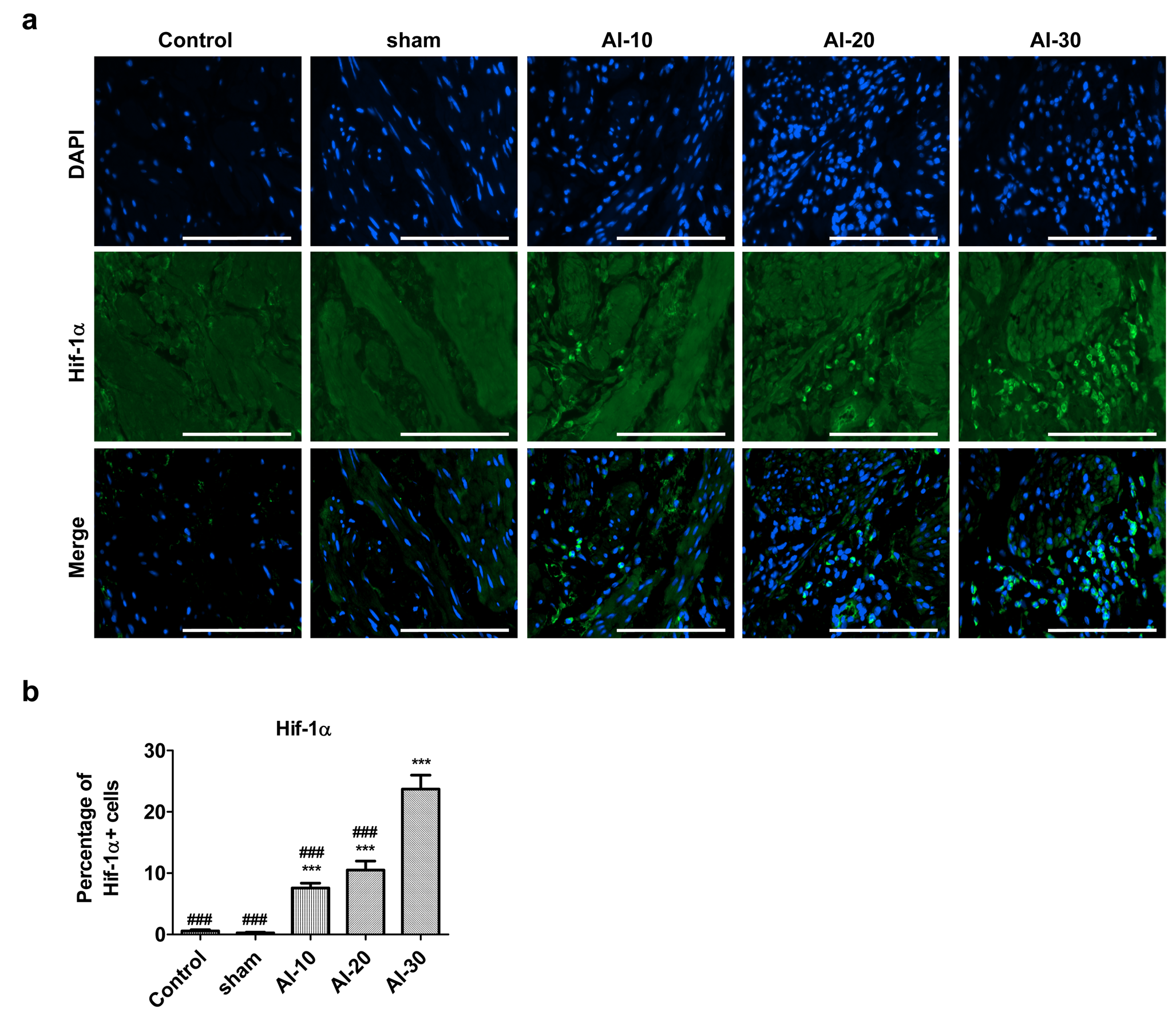


**Supplementary figure 3.** Increased expression of Hif-1α protein in the bladder tissues by the chronic bladder ischemia

Representative images (**a**) and quantification analysis (**b**) of immunofluorescence staining for Hif-1α protein (green) in the bladders in the rat of indicated groups (magnification ×400, scale bar=20 μm). In quantification analysis, the ratio of Hif-1α expressing cells to total cells (DAPI-stained nuclei) was calculated and percentage of Hif-1α staining cells are presented as mean ± SEM (n=10). **p <0.01, ***p <0.001 compared to the sham group; ###p <0.001 compared to the AI-30 group according to a one-way ANOVA with the Bonferroni post-hoc test.


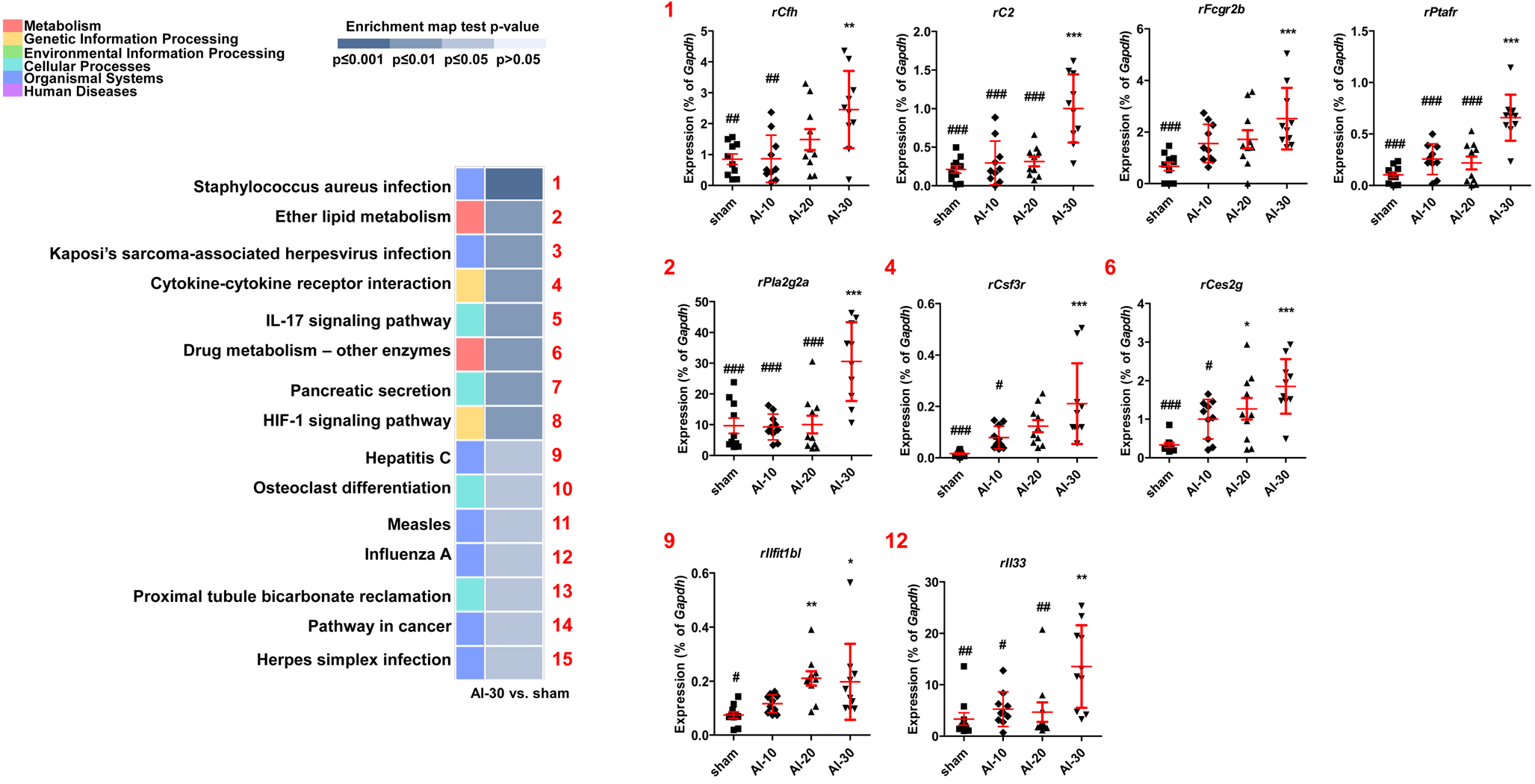


**Supplementary figure 4.** Expression analysis of genes characterized by progressive vascular endothelial damage

RQ-PCR analysis of genes which were significantly represented in the KEGG pathways with cut-off values of the enrichment map test p-value <0.05. Each gene was marked with the corresponding KEGG pathways with red colored numbers. Expression is presented as % *Gapdh* and shown as dot plot of mean ± SEM (n=10; *p <0.05, **p <0.01, ***p <0.001); KEGG pathway with the enrichment map test p-value and gene ontology were shown in right to RQ-PCR results.


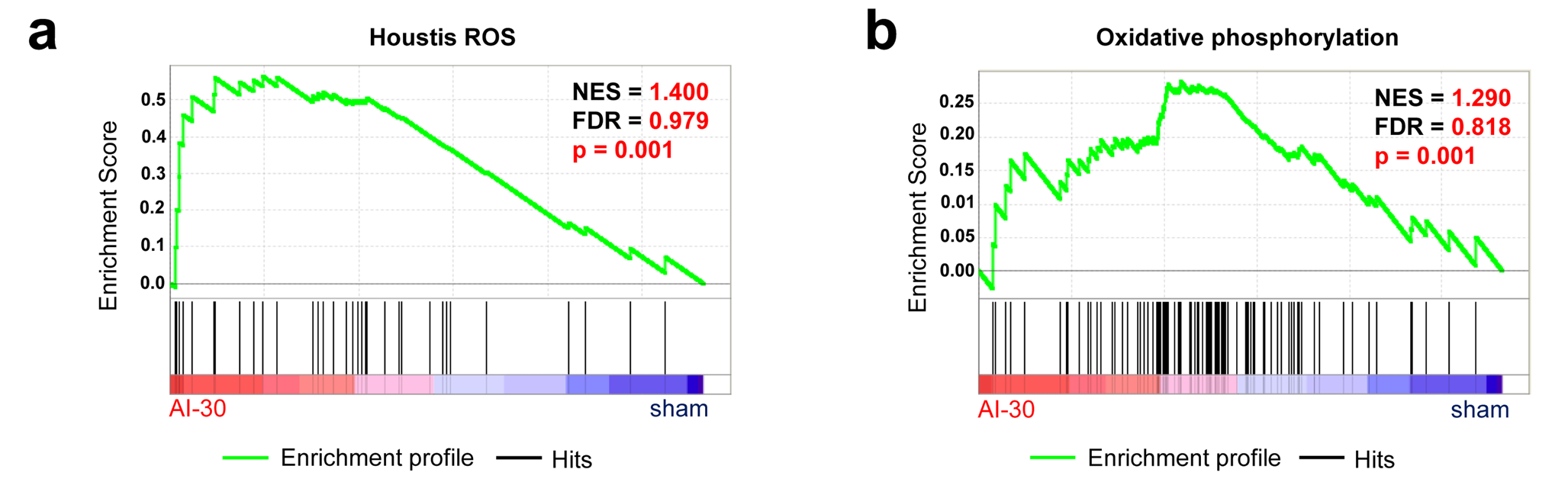


**Supplementary figure 5.** Gene Set Enrichment Analysis (GSEA) for gene sets of oxidative stress

(**a** and **b**) GSEA enrichment plots of gene sets involved in reactive oxygen species (ROS; **a**)^20^ and oxidative phosphorylation (curated by KEGG pathway; **b**) in a comparison of AI-30 versus sham transcriptomes; NES, normalized enrichment score; FDR, false discovery rate.


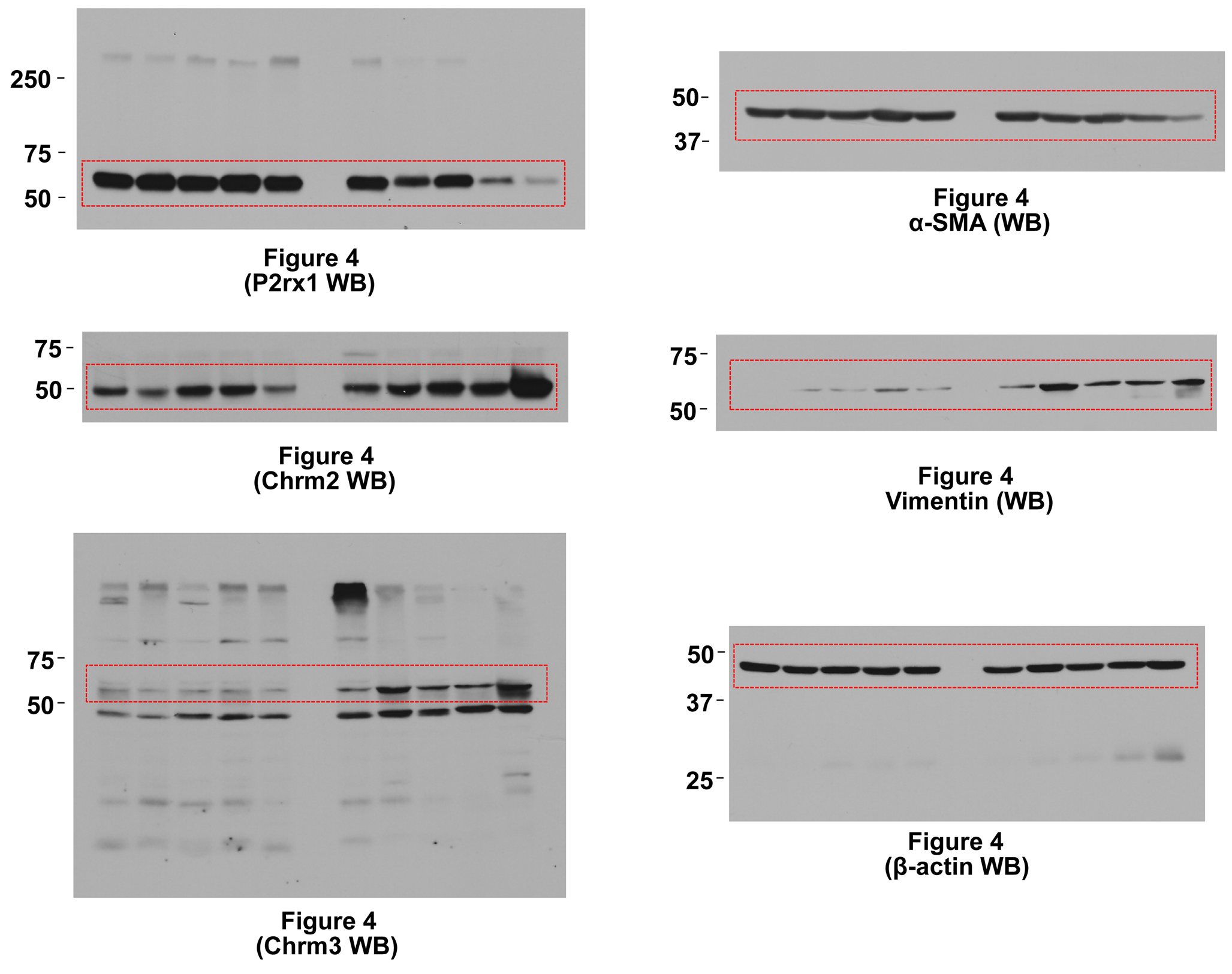


**Supplementary figure 6.** Uncropped western blots

Dotted red-line boxes indicate the cropped areas shown in the figures. In all the uncropped western blot images, the membranes were simultaneously or sequentially blotted with the indicated antibodies.
